# Anthrax lethal toxin exerts potent metabolic inhibition of the cardiovascular system

**DOI:** 10.1128/mbio.02160-24

**Published:** 2024-11-07

**Authors:** Jie Liu, Zehua Zuo, Rasem Fattah, Toren Finkel, Stephen H. Leppla, Shihui Liu

**Affiliations:** 1 Aging Institute of University of Pittsburgh and University of Pittsburgh Medical Center, Pittsburgh, Pennsylvania, USA; 2 Division of Cardiology, Department of Medicine, University of Pittsburgh School of Medicine, Pittsburgh, Pennsylvania, USA; 3 Division of Infectious Diseases, Department of Medicine, University of Pittsburgh School of Medicine, Pittsburgh, Pennsylvania, USA; 4 Microbial Pathogenesis Section, Laboratory of Parasitic Diseases, National Institute of Allergy and Infectious Diseases, National Institutes of Health, Bethesda, Maryland, USA; University of Michigan-Ann Arbor, Ann Arbor, Michigan, USA

**Keywords:** anthrax lethal toxin, *Bacillus anthracis*, c-Myc, ERK signaling, glycolysis, oxidative phosphorylation, metabolism

## Abstract

*Bacillus anthracis* causes anthrax through a combination of bacterial infection and toxemia. As a major virulence factor of *B. anthracis*, anthrax lethal toxin (LT) is a zinc-dependent metalloproteinase, exerting its cytotoxicity through proteolytic cleavage of the mitogen-activated protein kinase kinases, thereby shutting down the MAPK pathways. Anthrax lethal toxin induces host lethality mostly by targeting the cardiovascular system. Although the enzymatic activity and the molecular targets of LT have long been known, the detailed mechanisms underlying cellular/tissue/organ toxicity are still poorly understood. In this work, we sought to investigate the mechanism of LT-induced cellular damage in the cardiovascular system. We demonstrate for the first time that anthrax lethal toxin has potent inhibitory effects on the central metabolism of cardiomyocytes and endothelial cells. This is likely due to the observed downregulating of c-Myc expression through the toxin-induced inhibition of the ERK pathway. Since c-Myc is a master transcription factor controlling the expression of many rate-limiting metabolic enzymes in glycolysis and the tricarboxylic acid cycle, LT’s downregulation of c-Myc may lead to the observed bioenergetic collapse, particularly, in cardiomyocytes. Since cardiac cell contraction requires continuous production of large amounts of ATP, potent inhibition of the bioenergetics of cardiomyocytes would be incompatible with life. Thus, LT-induced lethality through targeting cardiomyocytes and endothelial cells appears to be a consequence of a bioenergetic collapse, likely due to the toxin’s potent inhibitory activity on the MEK-ERK-c-Myc-metabolic/bioenergetic axis within these target cells of cardiovascular system.

**IMPORTANCE:**

Anthrax lethal toxin (LT) is a major virulence factor of *Bacillus anthracis*, the causative pathogen of anthrax disease. Anthrax lethal toxin is a metalloproteinase that cleaves and inactivates MEKs, thereby shutting down MAPK pathways, leading to host mortality primarily through targeting of the cardiovascular system. However, the detailed mechanisms underlying the toxin’s cellular and tissue toxicity are still poorly understood. Here, we found that anthrax lethal toxin has potent inhibitory activity on glycolysis and oxidative phosphorylation of cardiomyocytes and endothelial cells. These effects appear to be the consequence of downregulation of c-Myc, a master transcription factor that controls many rate-limiting enzymes of glycolysis and the tricarboxylic acid cycle. With the high demand on energy for cardiac contraction, the potent inhibition of cardiomyocyte metabolism by LT would be incompatible with life. This work provides critical insights into why the cardiovascular system is the major *in vivo* target of LT-induced lethality.

## INTRODUCTION

*Bacillus anthracis* causes anthrax through a combination of bacterial infection and toxemia (1, 2). As the major virulence factors of *B. anthracis*, anthrax toxins play critical roles during multiple steps of the disease (1, 2). The three components of the anthrax exotoxins, that is, protective antigen (PA), lethal factor (LF), and edema factor (EF) are individually non-toxic, but they can pair to form the two toxins: lethal toxin (LT, composed of LF + PA) and edema toxin (ET, composed of EF + PA) (1, 2). Upon being secreted by *B. anthracis* during infection, PA binds to its cognate cellular receptors on the host target cell and is proteolytically processed by furin or furin-like proteases to form an oligomeric structure (PA heptamer or octamer) that can bind and translocate LF or EF into the cytosol of the target cell to exert their cytotoxic effects (3–5). EF is a calmodulin-dependent adenylyl cyclase that elevates intracellular cyclic AMP (cAMP) levels by converting ATP to cAMP, thereby causing cAMP-mediated diverse effects (e.g., tissue edema) (6). LF is a zinc-dependent metalloproteinase that cleaves and inactivates the mitogen-activated protein kinase kinases (MEKs) 1–4 and 6 (7–11). During infection, PA, LF, and EF are secreted in a roughly 25:5:1 ratio by *B. anthracis* (12, 13), thus making LT the most abundant toxin. The LF’s MEK cleavage events result in inactivation of the three mitogen-activated protein kinase (MAPK) pathways: the ERK pathway (through cleaving MEK1/2), the p38 pathway (through MEK3/6), and the JNK (Jun N-terminus kinase) pathway (through MEK4) (2, 14). MEK7 was initially reported to be a substrate of LF, but subsequent examination did not support this claim (8, 14), suggesting that the JNK pathway may only be partially affected by LT. These pathways play crucial roles in maintaining cell survival (e.g., the ERK pathway) and orchestrating responses to a diverse array of stresses (e.g., the p38 and JNK pathways).

We have previously generated and characterized a set of mice with cell-type-specific gain- or loss-of-function of the major cellular anthrax toxin receptor CMG2 (15–17). Analyzing these mice demonstrated that LT plays two critical roles in anthrax infection. At the initial stages of infection, LT impairs the host innate immune response, enabling the pathogen to survive the hostile environment in the host in order to establish a successful infection (15). This is believed to be attributable to the LT’s potent inhibitory effects on the production and secretion of pro-inflammatory cytokines and chemokines by innate immune cells (18, 19). At later stages, when high concentrations of LT are reached, LT can directly cause host death through targeting the cardiovascular system (17). Although the enzymatic activities and the molecular targets of LT have long been known, the detailed mechanisms underlying cellular/tissue/organ toxicity are still poorly understood. In this work, we sought to investigate the mechanism of LT-induced cellular damage in the cardiovascular system.

## RESULTS

### Cardiomyocytes and endothelial cells are *in vivo* targets of LT-induced lethality

**Fig 1:**
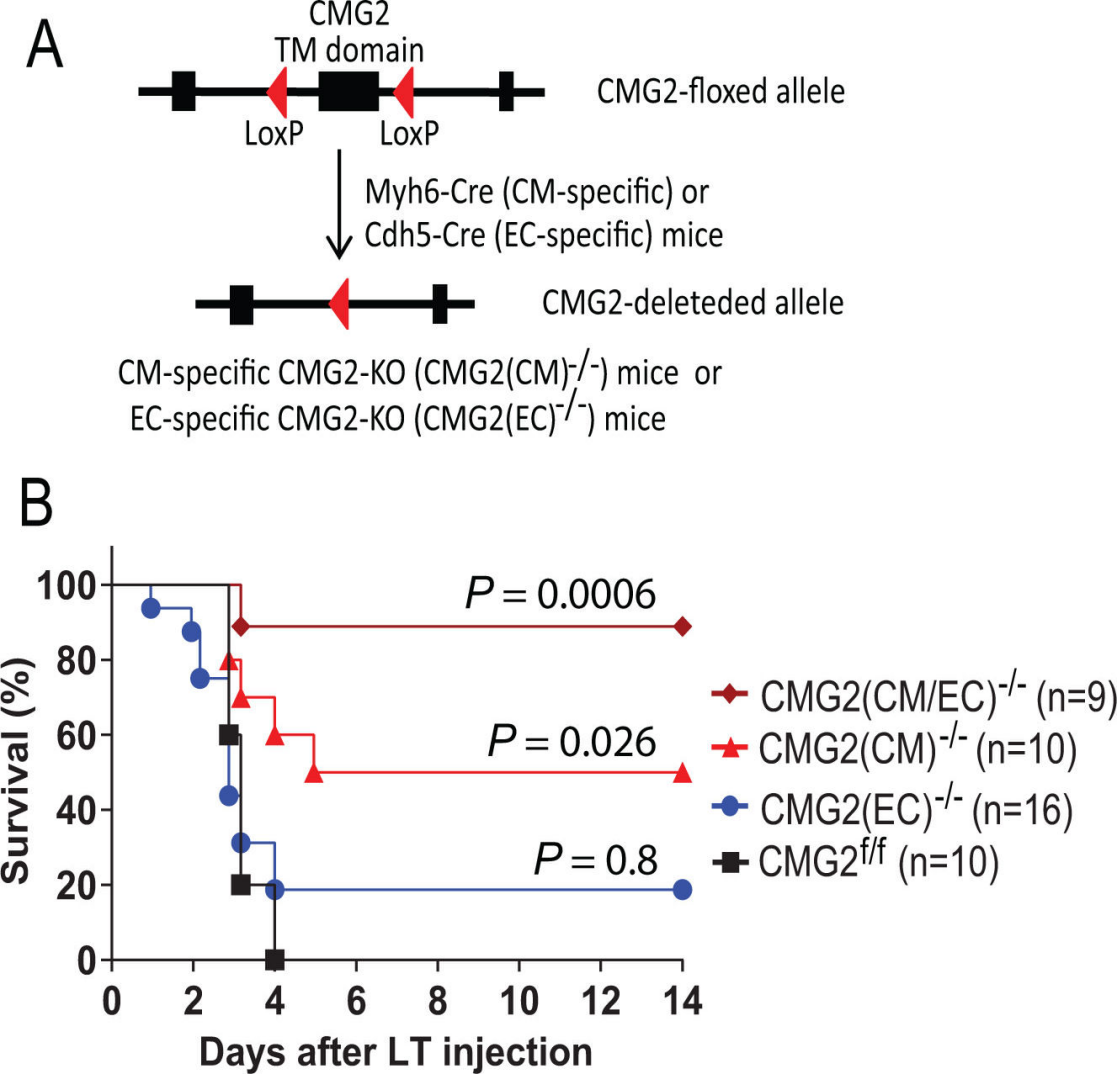
Cardiomyocytes and endothelial cells as major *in vivo* targets of anthrax lethal toxin. (**A**) Generation of CM- or EC-specific CMG2-KO mice by breeding CMG2-floxed mice with the corresponding tissue-specific Cre transgenic mice. Subsequent breeding resulted in the double tissue specific CMG2 KO [CMG2(CM/EC)^−/−^] mice. (**B**) Mice with indicated genotypes were injected (I.P.) with 100 µg LT (PA/LF, each 100 µg) and survival was monitored. Note, reduced susceptibility of CM-specific CMG2-KO [CMG2(CM)^−/−^] mice to the toxin was observed (*P* = 0.026, compared to the control CMG2^f/f^ mice). Although EC-specific CMG2-KO [CMG2(EC)^−/−^] mice remained sensitive to the toxin (*P* = 0.8), the mice [CMG2(CM/EC)^−/−^] with CMG2-KO in both ECs and CMs were nearly completely resistant to LT (*P* = 0.0006, log-rank test).

Cardiomyocytes and endothelial cells are the two major cell types of the cardiovascular system. We previously generated various cell-type-specific CMG2 receptor-KO (knockout) mice (15–17). Consistent with previous studies (17), here we showed that cardiomyocyte (CM)-specific CMG2-KO [CMG2(CM)^−/−^] mice have a significantly reduced susceptibility to LT when compared to their littermate control mice (Fig. 1). Interestingly, although knockout of the toxin receptor CMG2 in endothelial cells (ECs) [CMG2(EC)^−/−^] did not confer protection from LT challenge, we found that knockout of the toxin receptor from both CMs and ECs rendered the mice nearly completely resistant to LT (Fig. 1). These results demonstrate the essential roles of targeting CMs and, to a lesser extent, ECs in LT-induced lethality, and that the cardiovascular system is the major tissue target of LT-induced lethality.

### Profound anti-metabolic activity of LT on cardiomyocytes and endothelial cells

#### Studies on cardiomyocytes

**Fig 2:**
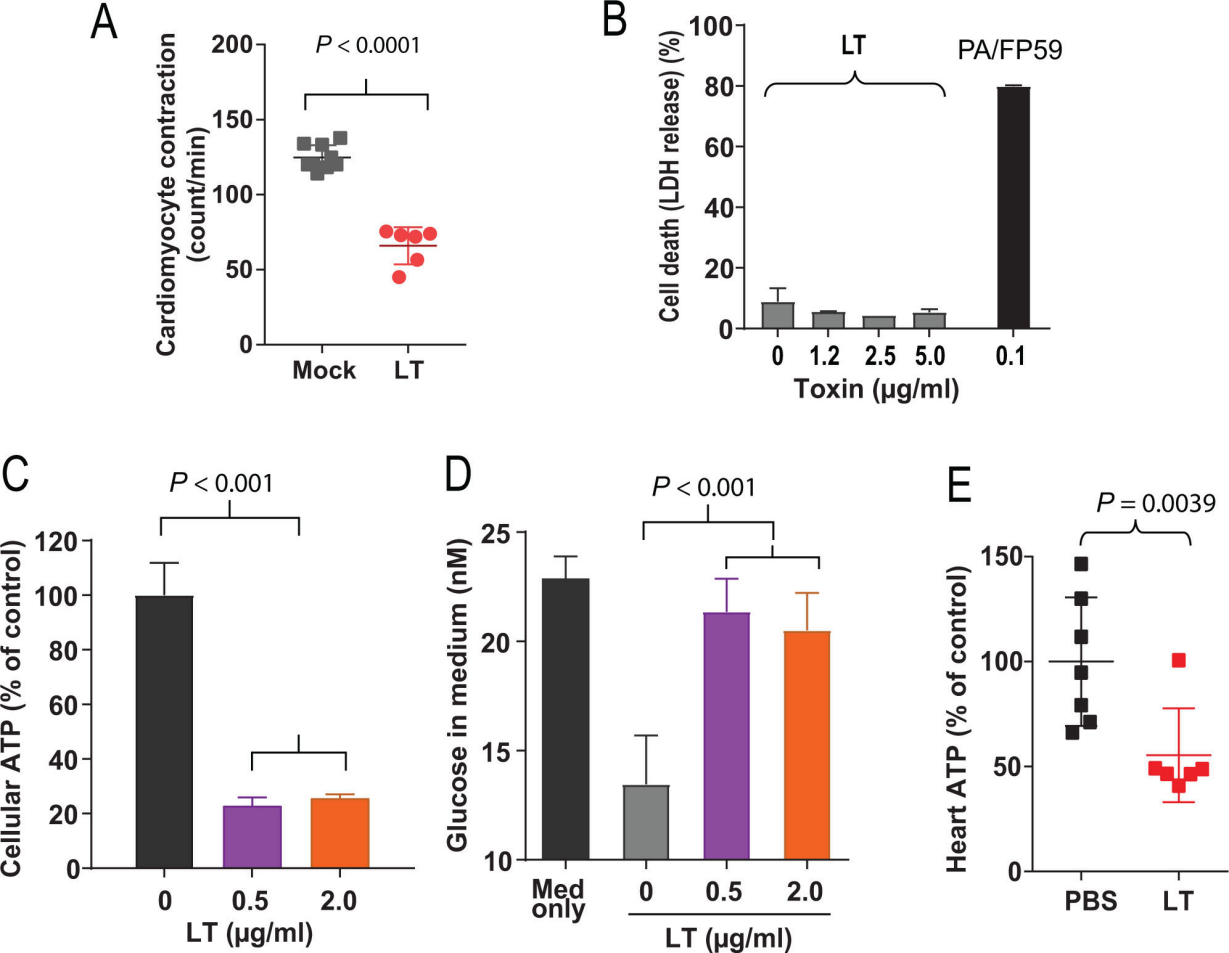
The potent inhibitory effects of anthrax lethal toxin on bioenergetics of cardiomyocytes. (**A**) Inhibition of the beating the beating activity of the cardiomyocytes. Beating CMs isolated from newborn mice were treated with LT (PA/LF, each 1 µg/mL) for 24 h, and contractions of the cells were recorded. Mean ± SD. Paired Student’s *t* test. (**B–D**) CMs were treated with the indicated concentrations of LT (1 µg/mL LT defined as 1 µg/mL of each PA and LF) for 24 h, then cell death (LDH assay) (**B**), cellular ATP levels (**C**), and medium glucose consumption (**D**) were measured. Of note, while PA/FP59 could efficiently kill CMs, LT did not induce CM death, but greatly reduced cellular ATP levels and glucose consumption. Data are shown as mean ± SD. (**E**) LT significantly reduced cardiac ATP levels. C57BL6 mice were injected with PBS or 100 µg LT (PA/LF, each 100 µg) (I.P.), and 24 h later, the hearts were collected and ATP levels measured. Data are shown as mean ± SD.

To investigate the molecular mechanisms of LT’s toxic effects on CMs, we isolated primary CMs from newborn mice following an established procedure (20), and treated the CMs with LT. Since the CMs we isolated were spontaneously beating cells, the most obvious LT’s effect we observed was a potent inhibitory activity of the toxin on CM contraction. While the untreated control cells underwent robust contraction with regular rhythms, the LT-treated CMs contracted irregularly with decreased frequency after 24-h incubation with LT (PA/LF, each 1 µg/mL, defined as 1 µg LT) (Fig. 2A; Videos S1 to S6). However, cytotoxicity experiments using lactate dehydrogenase (LDH) release assay showed that LT did not induce CM cell death (Fig. 2B; Videos S1 to S6). In contrast, when FP59 was used as an effector protein, PA/FP59 could efficiently kill CMs. FP59 is a fusion protein of LFn (N-terminal PA binding domain of LF) and the catalytic domain of *Pseudomonas aeruginosa* exotoxin A that kills all cells by ADP-ribosylation of eEF2 after PA-mediated delivery into the cytosol (21, 22). Because CM beating relies on energy, the above results suggest that LT may affect CMs’ bioenergetics. Indeed, we found that ATP levels of CMs were markedly decreased by LT (Fig. 2C). Further, glucose consumption of CMs was also remarkably diminished by LT (Fig. 2D). Importantly, the ATP levels of the hearts from the LT-challenged mice were also significantly reduced (Fig. 2E), demonstrating that LT affects ATP levels of CMs both *in vitro* and *in vivo*.

**Fig 3:**
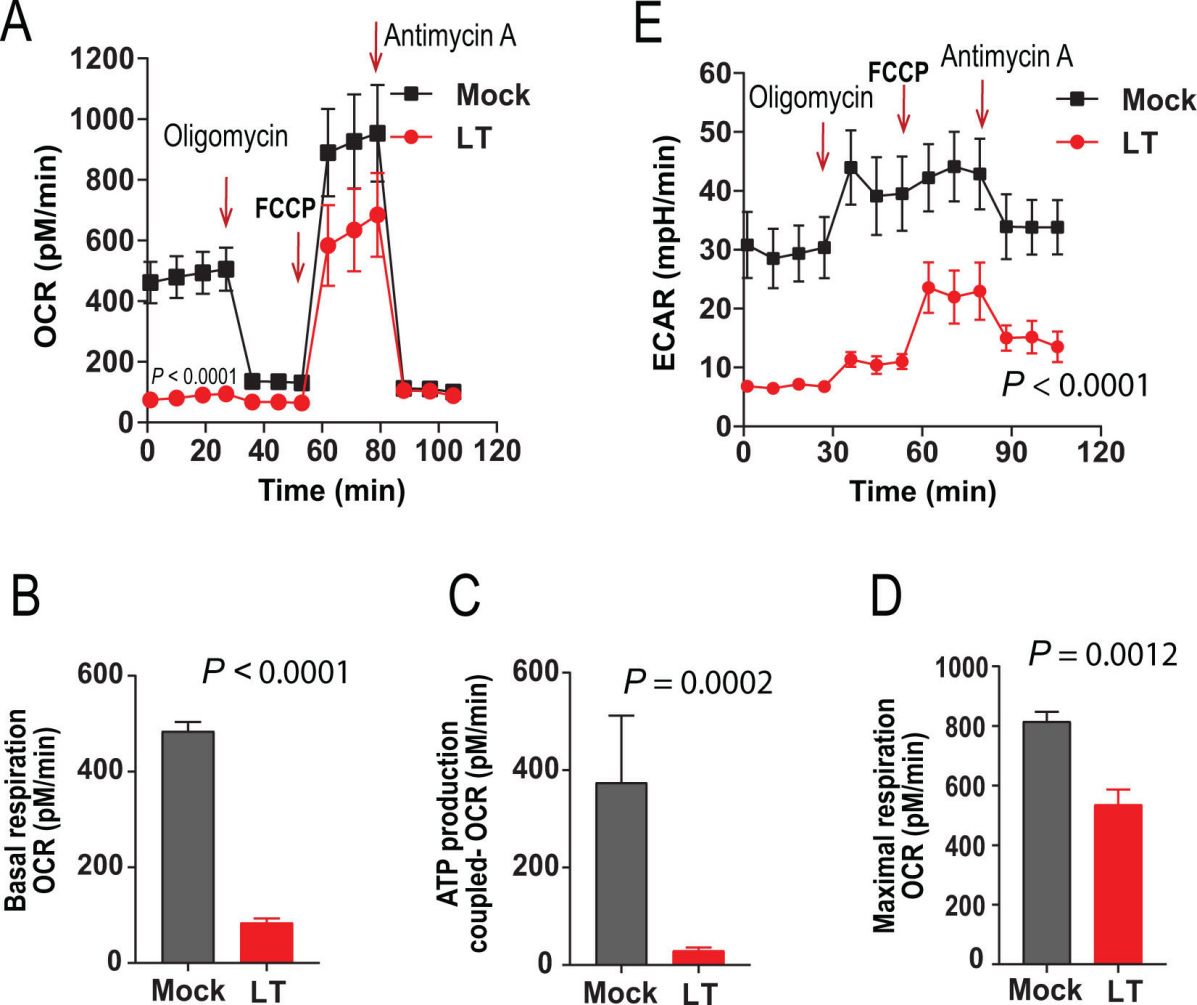
Potent inhibitory effects of anthrax lethal toxin on the metabolism of cardiomyocytes. (**A**) CMs were treated with or without LT (PA/LF, each 1 µg/mL) for 24 h, then oxygen consumption rates (OCRs) were measured using the Seahorse XF24 analyzer under basal conditions and following sequential additions of the ATP synthase inhibitor oligomycin (0.5 µg/mL), the oxidative phosphorylation uncoupler FCCP (1 µM), and complex III inhibitor antimycin A (0.5 µM). The OCR readings were normalized to 50 µg of total protein, mean ± SD. Paired Student’s *t* test, *P* < 0.0001. (**B–D**) Inhibitory effects of LT on CM’s basal oxygen consumption (**B**), ATP production-coupled oxygen consumption (**C**), and maximal respiration (**D**). (**E**) Extracellular acidification rate (ECAR) of CMs under the same conditions and treatments as in panel **A**. The ECAR readings were normalized to amounts of cells having 50 µg total protein, mean ± SD. Paired Student’s *t* test, *P* < 0.0001. Data are shown as means ± SD. Representative of three independent experiments with similar results.

To understand how LT affects the bioenergetics of CMs, we used Seahorse technology to examine the oxygen consumption rates (OCRs) and the extracellular acidification rates (ECARs) of the cells under basal conditions and following additions of the respiratory chain inhibitors (Seahorse Cell Mito Stress Test; see Materials and Methods) (Fig. 3). Interestingly, LT markedly inhibited CM’s basal oxygen consumption and ATP production-coupled oxygen consumption, defined as the difference between basal OCR and the OCR after the addition of oligomycin (Fig. 3A through C). The maximal respiration (MR), defined as the difference between the OCR following FCCP addition and the OCR following oligomycin addition, was also significantly decreased by LT treatment (Fig. 3A and D). Further, LT also greatly inhibited glycolytic activity under basal conditions (Fig. 3E). The upregulation of glycolytic activity, which attempts to compensate for diminished energy production during mitochondrial inhibition (by oligomycin and FCCP), was also compromised by the toxin (Fig. 3E). These results demonstrate that LT profoundly affects CM’s metabolism through inhibiting mitochondrial oxidative phosphorylation and glycolytic activity.

#### Studies on endothelial cells

**Fig 4:**
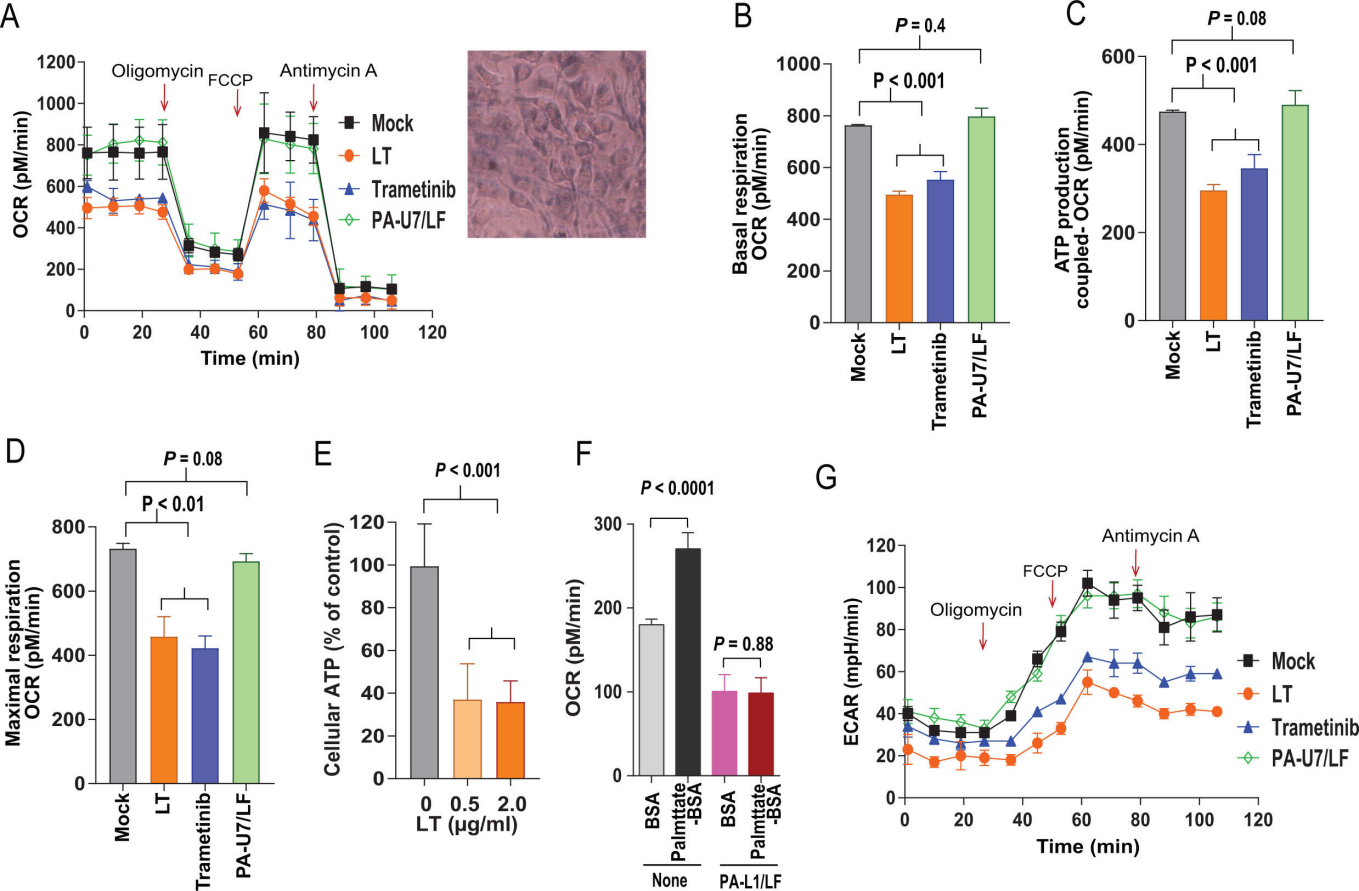
Potent inhibitory effects of anthrax lethal toxin on the metabolism of endothelial cells. (**A**) ECs were treated with or without LT (1 µg/mL each of PA and LF), PA-U7/LF (1 µg/mL each), or the MEK inhibitor Trametinib (0.2 µM) for 24 h, then the oxygen consumption rate (OCR) under the same conditions and treatments as in (Fig. 3) were measured. The OCR readings were normalized to 50 µg of total protein. Of note, the inhibitory effects of LT can be recapitulated by the MEK inhibitor Trametinib. Paired Student’s *t* test, *P* < 0.01. Means ± SD. Representative of three independent experiments with similar results. Insert, MTT-stained live ECs after 24 h LT treatment. (**B–D**) Inhibitory effects of LT on EC’s basal oxygen consumption (**B**), ATP production-coupled oxygen consumption (**C**), and maximal respiration (**D**). Means ± SD. (**E**) ECs were treated with the indicated concentrations of LT (1 µg/mL LT defined as 1 µg/mL of each PA and LF) for 24 h, then cellular ATP levels were measured. Means ± SD. (**F**) ECs treated with or without PA-L1/LF (1 µg/mL each, for 24 h) were incubated with palmitate-BSA or BSA and OCRs were determined. The OCR readings were normalized to 50 µg total protein, means ± SD. (**G**) Extracellular acidification rate (ECAR) under the same conditions and treatments as in panel **A** were measured. Means ± SD.

To evaluate whether LT has the similar inhibitory effects on the metabolism of ECs, primary ECs were isolated from mouse lungs following an established procedure (23, 24). As found for CMs, LT (1 µg/mL) could not directly kill ECs, but did significantly inhibit their mitochondrial oxidative phosphorylation activity, (Fig. 4A through D), accompanied by a reduction in cellular ATP production (Fig. 4E). In addition, while the fatty acid substrate palmitate-BSA could boost oxidative phosphorylation in untreated endothelial cells, this effect was also completely blocked by the toxin (Fig. 4F). These results were consistent with the inhibitory effects of the toxin on the tricarboxylic acid (TCA) cycle, leading to a block of acetyl-CoA (including that from fatty acid oxidation) consumption through oxidative phosphorylation. Further, LT also inhibited EC’s glycolytic activity under basal conditions and under mitochondrial stresses (Fig. 4G). Furthermore, the inhibitory effects on metabolism could be recapitulated by the MEK inhibitor Trametinib (Fig. 4A through D and G), indicating that the LT’s anti-metabolic effects are likely mediated through targeting of the MEK-ERK pathway. As an additional control, no inhibitory effects were observed when ECs were treated with LF plus PA-U7, a non-toxic PA variant that lacks furin protease cleavage site (Fig. 4A through D and G) (25, 26).

### Downregulation of *c-Myc* and key metabolic genes by LT

**Fig 5:**
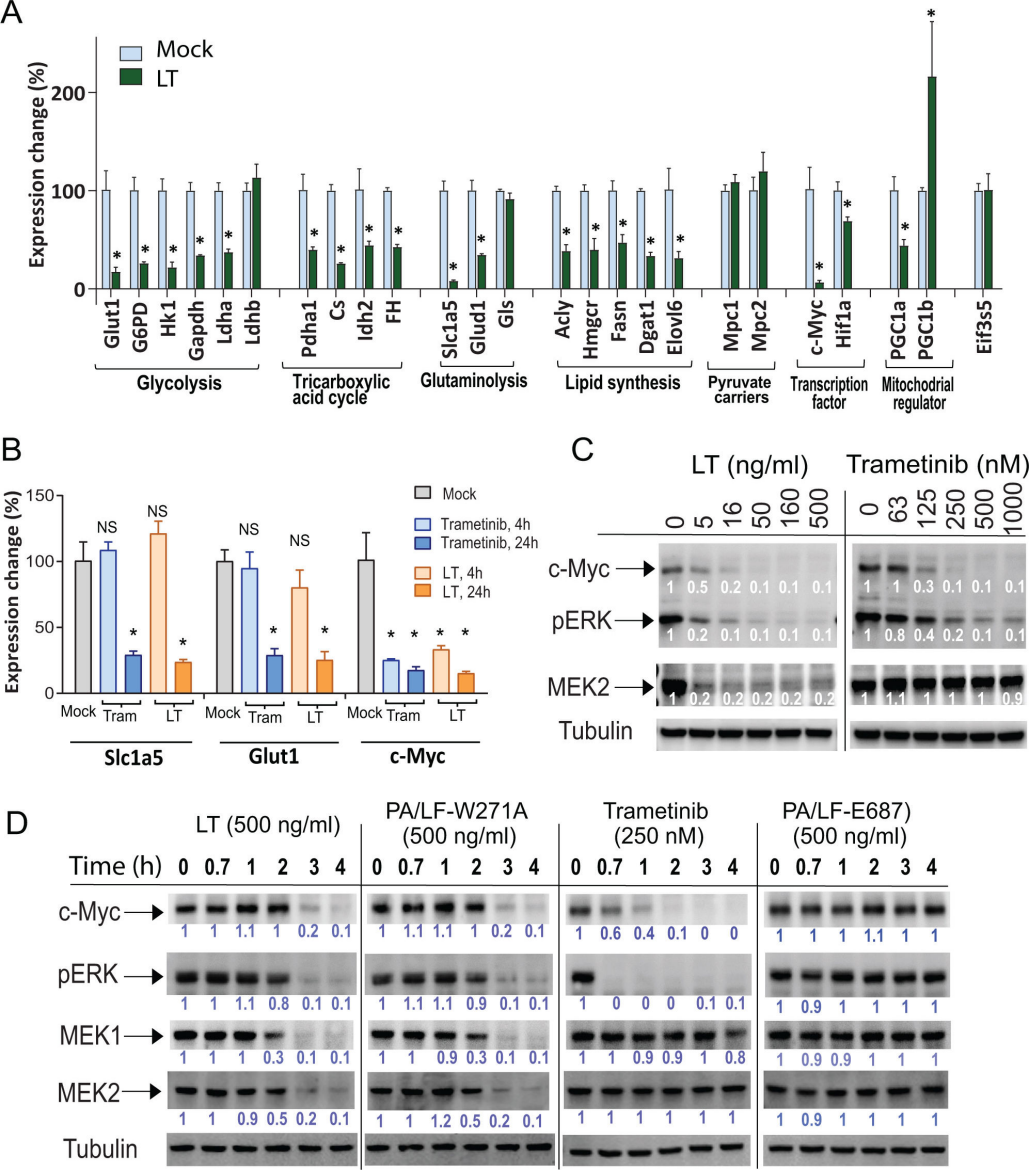
Downregulation of many key genes in endothelial cell metabolism following LT exposure. (**A**) Real-time PCR analyses demonstrate that many key genes in metabolism are down-regulated in endothelial cells treated with LT (PA/LF, each 1 µg/mL, 24 h). Note, c-Myc, a transcription factor with established roles in controlling the expression of key metabolic genes, was the most affected gene, with the expression level reduced to 6% of the untreated control. *Eif3s5* (Eukaryotic translation initiation factor 3, subunit 5) was used as an internal normalization control. Means ± SD. Representative of two independent experiments with similar results. (**B**) ECs were treated with Trametinib (0.2 µM) or LT (1 µg/mL) for 4 h or 24 h. Then, expression levels of *c-Myc* and *Slc1a5* (the most affected in (A)) were analyzed by real-time PCR. Mean ± SD. Student’s *t* test: *, *P* < 0.01. (**C**) MEK-ERK signaling controls c-Myc expression. ECs were incubated with various concentrations of LT (0–500 ng/mL, each of PA and LF)) or Trametinib (0–1000 nM) for 3 h. Then, cell lysates were prepared and analyzed by Western blotting using anti-phospho-ERK (pERK), anti-MEK2, and anti-cMyc antibodies. (**D**) ECs were incubated with LT (PA/LF, each 500 ng/mL) or Trametinib (200 nM) for various lengths of time. Then, cell lysates were prepared and analyzed by Western blotting using anti-phospho-ERK (pERK), anti-MEK1, anti-MEK2, and anti-cMyc antibodies. The relative protein band densities estimated using ImageJ were shown below each lane.

**Fig 6:**
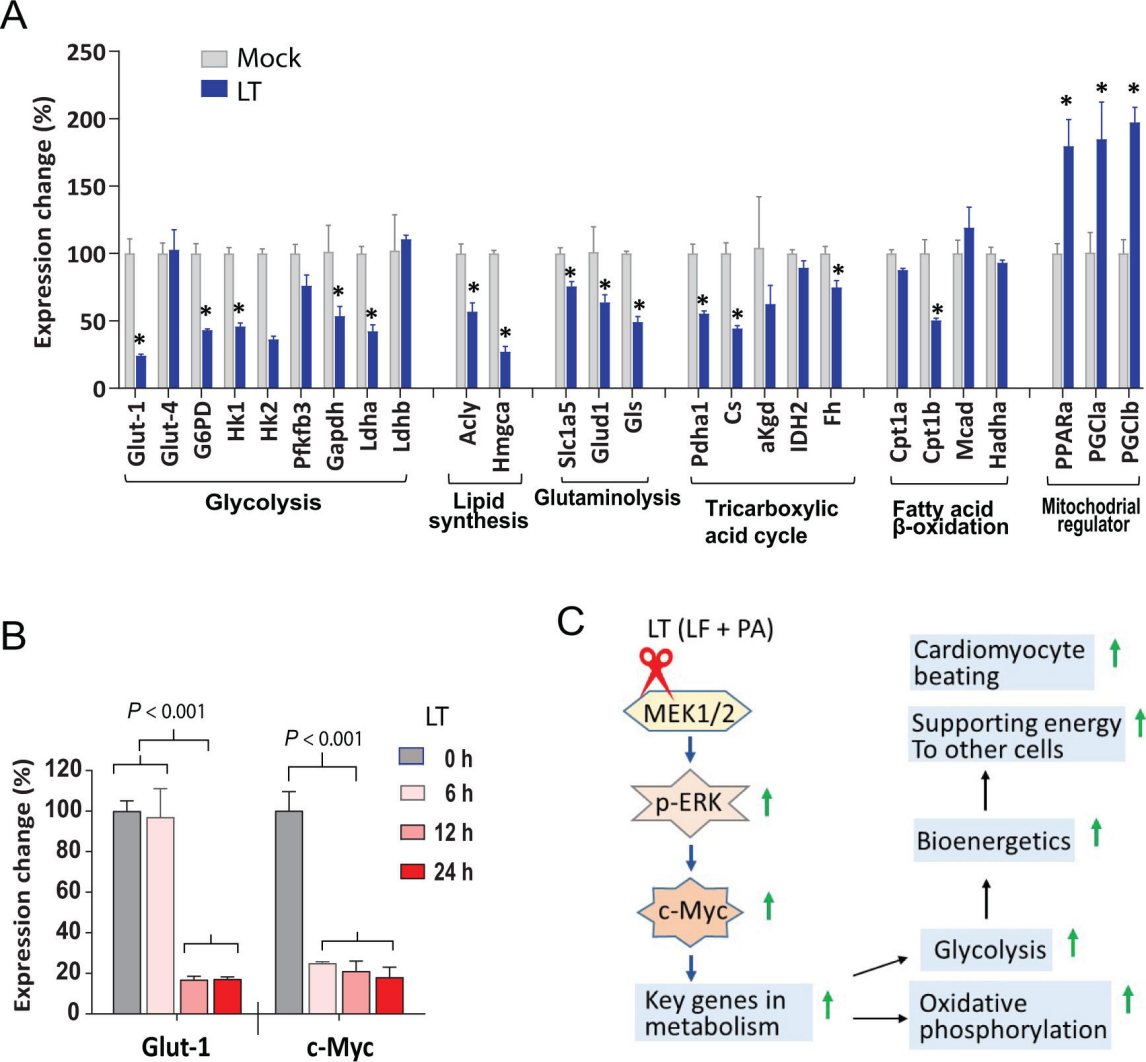
Downregulation of many key genes in metabolism in cardiomyocytes by LT (**A**) Real-time PCR analyses demonstrate that many key genes in metabolism are downregulated in cardiomyocytes treated with LT (PA/LF, each 1 µg/mL, 24 h). Representative of two independent experiments with similar results. (**B**) Real-time PCR analyses of *c-Myc* and *Glut1* (glucose transporter 1, the most affected in (A)) expression in mouse primary cardiomyocytes. CMs were incubated with LT (1 µg/mL) for various times (0, 6, 12, and 24 h) as indicated. Eif3s5 (LT-0 h) was used as an internal normalization control. Mean ± SD. Student’s *t* test: *, *P* < 0.01. (**C**) Graphical summary. Cancelation of the MEK-ERK-c-Myc-metabolic/bioenergetic axis by LT’s proteolytic inactivation of MEK1/2 results in bioenergetic collapse of cardiomyocytes and other cell types (e.g., ECs), potentially to be one of the major causes of LT-induced lethality.

To explore the mechanisms underlying LT’s inhibitory effects on metabolism, we surveyed the expression of key metabolic genes by real-time PCR analyses after treating ECs with the toxin for 24 h. Interestingly, many genes regulating glucose uptake, glycolysis, the TCA cycle, glutamine usage, as well as lipid synthesis were significantly downregulated by LT, whereas pyruvate carriers *Mpc1*, *Mpc2*, and some other mitochondrial genes were not affected (Fig. 5A). Transcriptional co-activator *PGC1b*, which has roles in promoting beta-oxidation and increasing mitochondrial number, was upregulated, reflecting a potential compensatory cellular response to the LT-induced energy deficit. Because the transcription factors c-Myc and HIF1α have established roles in regulating metabolism (27), we examined the effect of LT on their expression. Interestingly, while *Hif1α* was only modestly affected, *c-Myc* was markedly downregulated, with expression level reduced to 6% of the untreated controls within 24 h after toxin exposure. Interestingly, downregulation of *c-Myc* could be observed as soon as 4 h after LT treatment, while downregulation of other genes, such as *Glut1* and *Slc1a5* could only be seen at later time points (Fig. 5B). We further examined LT’s effect on c-Myc protein in ECs and found that LT markedly reduced c-Myc expression at the protein levels, and these effects could again be recapitulated by the MEK inhibitor Trametinib (Fig. 5C and D). As additional controls, while PA plus LF-E687C [a catalytic dead LF mutant (28)] had no effects, PA plus LF-W271A, a MEK1/2-specific LF variant (29), exhibited similar activity as seen for LT (Fig. 5D). The inhibitory effects of LT on the expression of some key metabolic genes and *c-Myc* were also observed in CMs (Fig. 6A). Similarly, downregulation of *c-Myc* could be observed early after LT treatment, while downregulation of other genes, such as *Glut1* could only be seen at later time points (Fig. 6B). Significant compensatory upregulation of the transcription factor *PPARa* and its co-activators *PGC1a* and *PGC1b* genes was also observed (Fig. 6A). Based on the fact that c-Myc is a master transcription factor having an established role in regulating many key genes in cellular metabolism (27, 30), it is likely that an important mechanism for the metabolic inhibition by LT is through its potent inhibitory effect on *c-Myc* expression.

In summary, our studies demonstrate that targeting the cardiovascular system plays a crucial role in LT-induced lethality. The lethality is largely due to impairing the function of CMs and, to a lesser extent, ECs. Mechanistically, this is likely due to the potent inhibitory effects of LT on the central metabolism and bioenergetics of these target cells, because of the markedly diminished expression of *c-Myc*. Therefore, disruption of the MEK-ERK-cMyc-bioenergetics axis is potentially one of the major causes of LT-induced lethality (Fig. 6C).

## DISCUSSION

As the major virulence factor of *B. anthracis*, anthrax LT can directly cause host death through targeting the cardiovascular system (17). Aligning well with this, here we showed that deletion of the major anthrax toxin receptor CMG2 only in endothelial cells does not make mice resistant to LT, and deletion of CMG2 only in cardiomyocytes makes mice partially resistant to LT. However, the mice with CMG2 deletion in both cardiomyocytes and endothelial cells were nearly completely resistant to a lethal dose of LT (e.g., a dose that killed all WT mice), demonstrating that CMs and, to a lesser extent, ECs are the cell types within the cardiovascular system responsible for LT-induced lethality. These results are in line with the results from previous studies demonstrating that LT negatively affects cardiac contraction (31), and that LT has the ability to disrupt endothelial cell-cell junction, barrier function, increasing vascular permeability (32–35).

To study how LT affects these cell types, we isolated primary cardiomyocytes and endothelial cells. Interestingly, LT did not greatly affect the viability of CMs and ECs, but dramatically inhibited their central metabolism, including glycolysis and oxidative phosphorylation. This was accompanied by downregulation of transcription of many key genes involved in cellular metabolism. All these appeared to be the consequence of downregulation of *c-Myc* by LT. c-Myc is a master transcription factor with an established role in promoting transcription of many key genes required for many rate-limiting steps of glycolysis, as well as the TCA cycle (27, 30, 36). LT’s potent inhibitory effect on c-Myc at both mRNA and protein levels was through its inactivation of the ERK pathway. This appears to also be true in many other cell types, such as tumor endothelial cells and many cancer cell lines (37). Therefore, anthrax lethal toxin induces cellular damage, at least partially, because of its potent anti-metabolic activity. Since cellular metabolism is essential for many cellular functions and it is estimated that vesicle trafficking and active transport processes consume approximately 50% of cellular ATP (38, 39), LT-induced-bioenergetic collapse may result in cargo proteins such as VE-cadherin cadherins failing to reach intercellular junctions, thus disrupting endothelial barrier function. Since cardiac contraction, which is essential for life of a whole organism, requires constant production of large amounts of ATP, potent inhibition of the metabolism of cardiomyocytes by LT would be life-threatening to the host. Although the LT-disruption of the ERK pathway and the metabolic inhibition occur in both cardiomyocytes and endothelial cells, we believe that inhibiting the heart beating through affecting bioenergetics metabolism of cardiomyocytes is likely to be a more important cause of cardiovascular system failure of the whole organism. Thus, LT-induced lethality through targeting cardiomyocytes and endothelial cells appears to be a consequence of the bioenergetic collapse due to the toxin’s potent inhibitory activity to the MEK-ERK-c-Myc-metabolic/bioenergetic axis within these target cells.

In summary, we demonstrate for the first time that anthrax lethal toxin has potent inhibitory effects on the central metabolism of cardiomyocytes and endothelial cells likely through potently downregulating *c-Myc*, a transcription factor that controls many key metabolic genes. This downregulation of c-Myc may lead to bioenergetic collapse, in particular, in cardiomyocytes. Since CMs have limited energetic reserves and depend on the production of large amounts of ATP for contraction, these studies provide critical insights into why the cardiovascular system is the major *in vivo* target of LT-induced lethality.

## MATERIALS AND METHODS

### Proteins and reagents

Recombinant PA and LF proteins were purified from supernatants of BH500, an avirulent, sporulation-defective, protease-deficient *B. anthracis* strain, as described previously (40, 41). FP59 is a fusion protein of LF amino acids 1–254 and the catalytic domain of *Pseudomonas aeruginosa* exotoxin A that kills cells by ADP-ribosylation of eEF2 after delivery to cytosol by PA (22). Trametinib (S2673) was from Selleckchem. MTT (3-[4,5-dimethylthiazol-2-yl]-2,5-diphenyltetrazolium bromide) was from Sigma (Atlanta, GA).

### Cells and cytotoxicity assay

All cultured cells were grown at 37°C in a 5% CO_2_ atmosphere. Mouse primary endothelial cells were isolated by following established protocols for mouse endothelial cell isolation (23, 24). Briefly, mouse lungs were digested with type I collagenase and plated on gelatin and collagen-coated flasks. The cells were then subjected to sequential negative sorting by magnetic beads coated with a sheep anti-rat antibody using a Fc Blocker (rat anti-mouse CD16/CD32, Cat. 553142, BD Pharmingen, San Diego, CA) to remove macrophages and positive sorting by magnetic beads using an anti-intermolecular adhesion molecule 2 (ICAM2 or CD102) antibody (Cat. 553326, BD Pharmingen) to isolate endothelial cells. Endothelial cells were cultured in DMEM supplemented with 20% fetal bovine serum, endothelial cell growth supplement (30 mg in 500 mL DMEM) (E2759, Sigma) from bovine neural tissue (containing both acidic and basic fibroblast growth factors), heparin (50 mg in 500 mL DMEM) (H3149-100 KU, Sigma).

Primary cardiomyocytes were isolated from newborn mice following an established protocol (20). Briefly, hearts were collected from 1 to 3 days old newborn mice (6–10 mice/litter) and digested with collagenase/dispase mixture. Then, the cells were pre-plated onto a 10-cm cell culture dish to allow fibroblasts and endothelial cells to adhere to the uncoated cell culture dish. Next, the cardiomyocytes in the medium were counted and plated with a density of 2 × 10^5^ onto collagen-coated 96-well plates for LDH release, cellular ATP measurement, or glucose consumption assay, or plated into 24-well XF24 tissue culture plates for OCR and ECAR measurements. Cellular ATP levels were measured using ATPlite 1step kit (PerkinElmer, Boston, MA), following the manufacturer’s manual. LDH release was measured by the LDH Assay Kit (Cytotoxicity) (Abcam, ab65393) following the manufacturer’s procedure. Glucose in the culture medium was measured using the Glucose Assay Lit (Abcam, ab65333) according to the manufacturer’s manual. Glucose consumption was represented as the differences between glucose levels in the medium only (without cells) and in conditioned medium of cultured cells.

For assessing the effects of LT on MEK-ERK-cMyc signaling, the cells were incubated with PA/LF for 3 h. Then, cell lysates were prepared in the modified RIPA lysis buffer containing protease inhibitors as described (26). Cell lysates were separated on SDS-PAGE gels, transferred onto nitrocellulose membranes, and analyzed by Western blotting using anti-MEK1 (#07-641, Upstate Technology), -MEK2 (#67410, Proteintech), anti-P-ERK (#4695, Cell Signaling), anti-c-Myc (Abcam), and anti-tubulin (#66031, Proteintech) antibodies. Relative protein abundance was quantified using ImageJ software (https://imagej.nih.gov/ij).

### Oxygen consumption rates and extracellular acidification rates

Metabolic activity of primary mouse cardiomyocytes and endothelial cells was assessed in an XF24 Extracellular Flux analyzer (Seahorse BioScience, North Billerica, MA). Cells grown to confluence in 24-well XF24 tissue culture plates were incubated with or without 1 µg/mL PA/LF (1 µg/mL each) (defined as 1 µg/mL LT) in pentaplicates for 24 h. Cells were changed into fresh unbuffered serum-free DMEM with 2 mM GlutaMax-1, 25 mM D-glucose (=4.5 g/L), pH 7.4, and equilibrated in the medium for 1 h, followed by Seahorse Cell Mito Stress test (https://www.agilent.com/cs/library/usermanuals/public/XF_Cell_Mito_Stress_Test_Kit_User_Guide.pdf). Real-time extracellular acidification rates (ECARs) and OCRs were obtained at 37°C under basal conditions and conditions following sequential additions of oligomycin (0.5 µM), FCCP (0.5 µM), and antimycin A (1 µM). ECARs and OCRs were normalized to 50 µg total protein in cell lysates. Oligomycin is a reagent that binds to the proton channel on the F_o_ component of ATP synthase, thereby blocking mitochondrial ATP synthesis, proton translocation, and oxygen consumption. ATP production-coupled OCR is calculated as the difference between basal OCR and OCR after the addition of oligomycin. FCCP is potent mitochondrial oxidative phosphorylation uncoupler that collapses the proton gradient and disrupts the mitochondrial membrane potential. Antimycin A is a complex III inhibitor, that inhibits mitochondrial respiration and enables the calculation of nonmitochondrial oxygen consumption.

For fatty acid oxidation assay, endothelial cells treated with or without LT (1 µg/mL) for 24 h were changed into fresh FAO assay medium (XF base medium [Seahorse BioScience, Cat. 102353-100] with 2.5 mM d-glucose, 0.5 mM carnitine, pH 7.4), and equilibrated in the medium for 1 h. Prior to starting XF assay, the medium was replaced with FAO assay medium containing 0.20 mM palmitate-BSA or 0.20 mM BSA (Seahorse BioScience, Cat. 102720-100).

### Gene expression

Endothelial cells or cardiomyocytes cultured in 12-well plates were treated LF/PA as indicated. Total RNA was then prepared using TRIzol reagent (Invitrogen, Carlsbad, CA). Single-strand cDNA was synthesized using reverse transcriptase reaction kit following the manufacturer’s manual (Invitrogen). Expression changes of the selected key genes involved in glucose uptake, glycolysis, TCA cycle, glutaminolysis, and lipid synthesis were analyzed by real-time PCR using SYBR Green PCR Mastermix. The primer sequences are shown in Table S1. A housekeeping gene *Eif3s5* (*TIF*), which largely remained constant among samples, was used as an internal control for relative gene expression comparisons. *C*_*t*_ values (cycle threshold) obtained from real-time PCR for each gene were first normalized to their internal *Eif3s5*’s *C*_*t*_ values, then expression levels were expressed as relative to their mock controls (set as 100%, the blue bars in Fig. 5A, gray bars in Fig. 6A).

### Mice

All animal studies were carried out in accordance with protocols approved by the University of Pittsburgh School of Medicine Animal Care and Use Committee. The mice having the CMG2 transmembrane (TM) domain-encoding exon 12 flanked by loxP sites (a “floxed” allele), namely, the CMG2^f/f^ mice (C57BL/6 background), were described previously (16). To generate mice having CMG2 deleted only in ECs, the CMG2^f/f^ mice were mated with *Cdh-Cre* transgenic mice (Cre-recombinase under the VE-cadherin promoter) (006137, the Jackson Laboratory, Bar Harbor, Maine, USA). EC-specific CMG2-KO mice [CMG2(EC)^−/−^] were obtained by the subsequent intercrossing of the resulting *CMG2^+/fl^/Cdh-Cre* mice. Similarly, to generate mice with CMG2 deleted in CMs [CMG2(CM)^−/−^], the CMG2^f/f^ mice were mated with *Myh6-Cre* (Cre-recombinase under CM-specific α myosin heavy polypeptide 6 promoter) (011038, the Jackson Laboratory) as described previously (17). The combined EC- and CM-specific CMG2-KO (CMG2(CM/EC)^−/−^) mice were obtained from the breeding of CMG2(EC)^−/−^) and CMG2(CM)^−/−^) mice. All these mice had a pure C57BL/6 background. In LT challenge experiments, 10- to 12-week-old male and female mice with indicated genotypes were injected with 100 µg LT (100 µg PA + 100 µg LF) in 0.5 mL phosphate-buffered saline (PBS) intraperitoneally and were monitored twice daily for 2 weeks post-challenge for signs of malaise or mortality. Genotyping was performed by PCR using mouse ear DNA described previously (17).

To extract and measure ATP levels of the hearts from LT-challenged mice, the mice were treated with or without 100 µg LT (I.P.). The mice were killed after 24 h and hearts were quickly collected. Then, ~0.3 g of heart tissue from each mouse was homogenized in 3 mL of ice-cold phenol-TE on ice (42). The homogenate (1 mL) was mixed well with 200 µL of chloroform and 150 µL of deionized water and centrifuged at 10,000 × *g* for 5 min. After this, the upper aqueous phase (50 µL) was collected, diluted, and assessed for ATP levels using ATPlite 1step (PerkinElmer, 6016731). ATP levels of the hearts were normalized to tissue-wet weight. ATP levels of the hearts from the LT-treated mice are presented as percentages of heart ATP levels in the untreated mice.

### Statistics

Statistical significances of differences were calculated using unpaired or paired two-tailed Student’s *t* test or one-way ANOVA analysis when more than two groups were compared. Survival curves were compared using the log-rank test. *P* < 0.05 is considered as significant difference.

## Data Availability

All data supporting the findings of this study are available within the paper and its supplemental material.

## SUPPLEMENTAL MATERIAL

Supplemental video legends mbio.02160-24-s0001.docx Legends for Videos S1-S6.

Table S1 mbio.02160-24-s0002.docx Primers used for real-time PCR analyses of key genes in metabolism.

Video S1 mbio.02160-24-s0003.mp4 Mouse cardiomyocytes without LT treatment.

Video S2 mbio.02160-24-s0004.mp4 Mouse cardiomyocytes without LT treatment.

Video S3 mbio.02160-24-s0005.mp4 Mouse cardiomyocytes without LT treatment.

Video S4 mbio.02160-24-s0006.mp4 LT-treated mouse cardiomyocytes contracted irregularly with decreased frequency after 24-h incubation with LT.

Video S5 mbio.02160-24-s0007.mp4 LT-treated mouse cardiomyocytes contracted irregularly with decreased frequency after 24-h incubation with LT.

Video S6 mbio.02160-24-s0008.mp4 LT-treated mouse cardiomyocytes contracted irregularly with decreased frequency after 24-h incubation with LT.

ASM does not own the copyrights to Supplemental Material that may be linked to, or accessed through, an article. The authors have granted ASM a non-exclusive, world-wide license to publish the Supplemental Material files. Please contact the corresponding author directly for reuse.

## References

[1] Liu S, Moayeri M, Leppla SH. 2014. Anthrax lethal and edema toxins in anthrax pathogenesis. Trends Microbiol 22:317–325. doi:10.1016/j.tim.2014.02.012 24684968 PMC4041834

[2] Moayeri M, Leppla SH, Vrentas C, Pomerantsev AP, Liu S. 2015. Anthrax pathogenesis. Annu Rev Microbiol 69:185–208. doi:10.1146/annurev-micro-091014-104523 26195305

[3] Kintzer AF, Thoren KL, Sterling HJ, Dong KC, Feld GK, Tang II, Zhang TT, Williams ER, Berger JM, Krantz BA. 2009. The protective antigen component of anthrax toxin forms functional octameric complexes. J Mol Biol 392:614–629. doi:10.1016/j.jmb.2009.07.037 19627991 PMC2742380

[4] Phillips DD, Fattah RJ, Crown D, Zhang Y, Liu S, Moayeri M, Fischer ER, Hansen BT, Ghirlando R, Nestorovich EM, Wein AN, Simons L, Leppla SH, Leysath CE. 2013. Engineering anthrax toxin variants that exclusively form octamers and their application to targeting tumors. J Biol Chem 288:9058–9065. doi:10.1074/jbc.M113.452110 23393143 PMC3610978

[5] Collier RJ. 2009. Membrane translocation by anthrax toxin. Mol Aspects Med 30:413–422. doi:10.1016/j.mam.2009.06.003 19563824 PMC2783560

[6] Leppla SH. 1982. Anthrax toxin edema factor: a bacterial adenylate cyclase that increases cyclic AMP concentrations of eukaryotic cells. Proc Natl Acad Sci U S A 79:3162–3166. doi:10.1073/pnas.79.10.3162 6285339 PMC346374

[7] Duesbery NS, Webb CP, Leppla SH, Gordon VM, Klimpel KR, Copeland TD, Ahn NG, Oskarsson MK, Fukasawa K, Paull KD, Vande Woude GF. 1998. Proteolytic inactivation of MAP-kinase-kinase by anthrax lethal factor. Science 280:734–737. doi:10.1126/science.280.5364.734 9563949

[8] Lee CS, Dykema KJ, Hawkins DM, Cherba DM, Webb CP, Furge KA, Duesbery NS. 2011. MEK2 is sufficient but not necessary for proliferation and anchorage-independent growth of SK-MEL-28 melanoma cells. PLoS One 6:e17165. doi:10.1371/journal.pone.0017165 21365009 PMC3041822

[9] Vitale G, Pellizzari R, Recchi C, Napolitani G, Mock M, Montecucco C. 1998. Anthrax lethal factor cleaves the N-terminus of MAPKKs and induces tyrosine/threonine phosphorylation of MAPKs in cultured macrophages. Biochem Biophys Res Commun 248:706–711. doi:10.1006/bbrc.1998.9040 9703991

[10] Vitale G, Bernardi L, Napolitani G, Mock M, Montecucco C. 2000. Susceptibility of mitogen-activated protein kinase kinase family members to proteolysis by anthrax lethal factor. BiochemJ 352:739–745. doi:10.1042/bj3520739 11104681 PMC1221512

[11] Zuo Z, Liu J, Sun Z, Silverstein R, Zou M, Finkel T, Bugge TH, Leppla SH, Liu S. 2022. A potent tumor-selective ERK pathway inactivator with high therapeutic index. PNAS Nexus 1:pgac104. doi:10.1093/pnasnexus/pgac104 35899070 PMC9308561

[12] Mabry R, Brasky K, Geiger R, Carrion R, Hubbard GB, Leppla SH, Patterson JL, Georgiou G, Iverson BL. 2006. Detection of anthrax toxin in the serum of animals infected with Bacillus anthracis by using engineered immunoassays. Clin Vaccine Immunol 13:671–677. doi:10.1128/CVI.00023-06 16760326 PMC1489546

[13] Molin FD, Fasanella A, Simonato M, Garofolo G, Montecucco C, Tonello F. 2008. Ratio of lethal and edema factors in rabbit systemic anthrax. Toxicon 52:824–828. doi:10.1016/j.toxicon.2008.08.011 18812184

[14] Ouyang W, Guo P, Fang H, Frucht DM. 2017. Anthrax lethal toxin rapidly reduces c-Jun levels by inhibiting c-Jun gene transcription and promoting c-Jun protein degradation. J Biol Chem 292:17919–17927. doi:10.1074/jbc.M117.805648 28893904 PMC5663889

[15] Liu S, Miller-Randolph S, Crown D, Moayeri M, Sastalla I, Okugawa S, Leppla SH. 2010. Anthrax toxin targeting of myeloid cells through the CMG2 receptor is essential for establishment of Bacillus anthracis infections in mice. Cell Host Microbe 8:455–462. doi:10.1016/j.chom.2010.10.004 21075356 PMC3032408

[16] Liu S, Crown D, Miller-Randolph S, Moayeri M, Wang H, Hu H, Morley T, Leppla SH. 2009. Capillary morphogenesis protein-2 is the major receptor mediating lethality of anthrax toxin in vivo. Proc Natl Acad Sci USA 106:12424–12429. doi:10.1073/pnas.0905409106 19617532 PMC2718377

[17] Liu S, Zhang Y, Moayeri M, Liu J, Crown D, Fattah RJ, Wein AN, Yu ZX, Finkel T, Leppla SH. 2013. Key tissue targets responsible for anthrax-toxin-induced lethality. Nature New Biol 501:63–68. doi:10.1038/nature12510 PMC4080305 23995686

[18] Cleret-Buhot A, Mathieu J, Tournier JN, Quesnel-Hellmann A. 2012. Both lethal and edema toxins of Bacillus anthracis disrupt the human dendritic cell chemokine network. PLoS One 7:e43266. doi:10.1371/journal.pone.0043266 22937027 PMC3427382

[19] Tournier J-N, Rossi Paccani S, Quesnel-Hellmann A, Baldari CT. 2009. Anthrax toxins: a weapon to systematically dismantle the host immune defenses. Mol Aspects Med 30:456–466. doi:10.1016/j.mam.2009.06.002 19560486

[20] Ehler E, Moore-Morris T, Lange S. 2013. Isolation and culture of neonatal mouse cardiomyocytes. J Vis Exp 79:50154. doi:10.3791/50154 PMC3857885 24056408

[21] Arora N, Klimpel KR, Singh Y, Leppla SH. 1992. Fusions of anthrax toxin lethal factor to the ADP-ribosylation domain of Pseudomonas exotoxin A are potent cytotoxins which are translocated to the cytosol of mammalian cells. J Biol Chem 267:15542–15548. 1639793

[22] Liu S, Bachran C, Gupta P, Miller-Randolph S, Wang H, Crown D, Zhang Y, Wein AN, Singh R, Fattah R, Leppla SH. 2012. Diphthamide modification on eukaryotic elongation factor 2 is needed to assure fidelity of mRNA translation and mouse development. Proc Natl Acad Sci U S A 109:13817–13822. doi:10.1073/pnas.1206933109 22869748 PMC3427129

[23] Liu S, Liu J, Ma Q, Cao L, Fattah RJ, Yu Z, Bugge TH, Finkel T, Leppla SH. 2016. Solid tumor therapy by selectively targeting stromal endothelial cells. Proc Natl Acad Sci USA 113:E4079–E4087. doi:10.1073/pnas.1600982113 27357689 PMC4948345

[24] Reynolds LE, Hodivala-Dilke KM. 2006. Primary mouse endothelial cell culture for assays of angiogenesis. Methods Mol Med 120:503–509. doi:10.1385/1-59259-969-9:503 16491622

[25] Liu S, Bugge TH, Leppla SH. 2001. Targeting of tumor cells by cell surface urokinase plasminogen activator-dependent anthrax toxin. J Biol Chem 276:17976–17984. doi:10.1074/jbc.M011085200 11278833

[26] Liu S, Leung HJ, Leppla SH. 2007. Characterization of the interaction between anthrax toxin and its cellular receptors. Cell Microbiol 9:977–987. doi:10.1111/j.1462-5822.2006.00845.x 17381430 PMC2459336

[27] Stine ZE, Walton ZE, Altman BJ, Hsieh AL, Dang CV. 2015. MYC, metabolism, and cancer. Cancer Discov 5:1024–1039. doi:10.1158/2159-8290.CD-15-0507 26382145 PMC4592441

[28] Gupta M, Alam S, Bhatnagar R. 2007. Catalytically inactive anthrax toxin(s) are potential prophylactic agents. Vaccine (Auckl) 25:8410–8419. doi:10.1016/j.vaccine.2007.09.063 17980467

[29] Goldberg AB, Cho E, Miller CJ, Lou HJ, Turk BE. 2017. Identification of a substrate-selective exosite within the metalloproteinase anthrax lethal factor. J Biol Chem 292:814–825. doi:10.1074/jbc.M116.761734 27909054 PMC5247655

[30] Hsieh AL, Walton ZE, Altman BJ, Stine ZE, Dang CV. 2015. MYC and metabolism on the path to cancer. Semin Cell Dev Biol 43:11–21. doi:10.1016/j.semcdb.2015.08.003 26277543 PMC4818970

[31] Kandadi MR, Yu X, Frankel AE, Ren J. 2012. Cardiac-specific catalase overexpression rescues anthrax lethal toxin-induced cardiac contractile dysfunction: role of oxidative stress and autophagy. BMC Med 10:134. doi:10.1186/1741-7015-10-134 23134810 PMC3520786

[32] Bolcome RE III, Sullivan SE, Zeller R, Barker AP, Collier RJ, Chan J. 2008. Anthrax lethal toxin induces cell death-independent permeability in zebrafish vasculature. Proc Natl Acad Sci USA 105:2439–2444. doi:10.1073/pnas.0712195105 18268319 PMC2268155

[33] Warfel JM, D’Agnillo F. 2011. Anthrax lethal toxin-mediated disruption of endothelial VE-cadherin is attenuated by inhibition of the Rho-associated kinase pathway. Toxins (Basel) 3:1278–1293. doi:10.3390/toxins3101278 22069696 PMC3210462

[34] Warfel JM, Steele AD, D’Agnillo F. 2005. Anthrax lethal toxin induces endothelial barrier dysfunction. Am J Pathol 166:1871–1881. doi:10.1016/S0002-9440(10)62496-0 15920171 PMC1602427

[35] Langer M, Duggan ES, Booth JL, Patel VI, Zander RA, Silasi-Mansat R, Ramani V, Veres TZ, Prenzler F, Sewald K, Williams DM, Coggeshall KM, Awasthi S, Lupu F, Burian D, Ballard JD, Braun A, Metcalf JP. 2012. Bacillus anthracis lethal toxin reduces human alveolar epithelial barrier function. Infect Immun 80:4374–4387. doi:10.1128/IAI.01011-12 23027535 PMC3497415

[36] Dang CV. 2013. MYC, metabolism, cell growth, and tumorigenesis. Cold Spring Harb Perspect Med 3:a014217. doi:10.1101/cshperspect.a014217 23906881 PMC3721271

[37] Zuo Z, Liu J, Sun Z, Cheng YW, Ewing M, Bugge TH, Finkel T, Leppla SH, Liu S. 2023. ERK and c-Myc signaling in host-derived tumor endothelial cells is essential for solid tumor growth. Proc Natl Acad Sci U S A 120:e2211927120. doi:10.1073/pnas.2211927120 36574698 PMC9910475

[38] Puurand M, Tepp K, Klepinin A, Klepinina L, Shevchuk I, Kaambre T. 2018. Intracellular energy-transfer networks and high-resolution respirometry: a convenient approach for studying their function. Int J Mol Sci 19:2933. doi:10.3390/ijms19102933 30261663 PMC6213097

[39] Cui L, Li H, Xi Y, Hu Q, Liu H, Fan J, Xiang Y, Zhang X, Shui W, Lai Y. 2022. Vesicle trafficking and vesicle fusion: mechanisms, biological functions, and their implications for potential disease therapy. Mol Biomed 3:29. doi:10.1186/s43556-022-00090-3 36129576 PMC9492833

[40] Gupta PK, Moayeri M, Crown D, Fattah RJ, Leppla SH. 2008. Role of N-terminal amino acids in the potency of anthrax lethal factor. PLoS One 3:e3130. doi:10.1371/journal.pone.0003130 18769623 PMC2518864

[41] Pomerantsev AP, Pomerantseva OM, Moayeri M, Fattah R, Tallant C, Leppla SH. 2011. A Bacillus anthracis strain deleted for six proteases serves as an effective host for production of recombinant proteins. Protein Expr Purif 80:80–90. doi:10.1016/j.pep.2011.05.016 21827967 PMC3183367

[42] Chida J, Kido H. 2014. Extraction and quantification of adenosine triphosphate in mammalian tissues and cells. Methods Mol Biol 1098:21–32. doi:10.1007/978-1-62703-718-1_2 24166365

