# Supplementary material for: Anthrax lethal toxin exerts potent metabolic inhibition of the cardiovascular system
Source: mBio. 2024 Nov 7;15(12):e02160-24. doi: 10.1128/mbio.02160-24 (PMC11633152; doi:10.1128/mbio.02160-24)
Supplement: Supplemental video legends — Legends for Videos S1-S6. [file mbio.02160-24-s0001.docx]

**Supplemental video legends**

**Supplemental video 1**. Regularly contracting mouse cardiomyocytes without LT treatment.

**Supplemental video 2**. Regularly contracting mouse cardiomyocytes without LT treatment.

**Supplemental video 3**. Regularly contracting mouse cardiomyocytes without LT treatment.

**Supplemental video 4**. LT-treated mouse cardiomyocytes contracted irregularly with decreased frequency after 24 h-incubation with LT.

**Supplemental video 5**. LT-treated mouse cardiomyocytes contracted irregularly with decreased frequency after 24 h-incubation with LT.

**Supplemental video 6**. LT-treated mouse cardiomyocytes contracted irregularly with decreased frequency after 24 h-incubation with LT.
