# Supplementary material for: Anthrax lethal toxin exerts potent metabolic inhibition of the cardiovascular system
Source: mBio. 2024 Nov 7;15(12):e02160-24. doi: 10.1128/mbio.02160-24 (PMC11633152; doi:10.1128/mbio.02160-24)
Supplement: Table S1 — Primers used for real-time PCR analyses of key genes in metabolism. [file mbio.02160-24-s0002.docx]

**Table S1**. Primers used for real-time PCR analyses of key genes in metabolism (Related to Figures 5 and 6)

| Gene name | Primer name | Sequence | Note |
| --- | --- | --- | --- |
| *Glut1* | GLUT1-F | 5’-AGCAGTGCTCGGATCACTGCAGTTC-3’ | Glucose uptake |
|  | GLUT1-R | 5’-AACAGGTTCATCATCAGCATGGAGT-3’ |  |
| *Glut4* | GLUT4-F | 5’-CGAGTGACTGGAACACTGGTCCTA-3’ | Glucose uptake |
|  | GLUT4-R | 5’-GCCAATGAGAAAGGAAGAGATCAT-3’ |  |
| *G6PD* | G6PD-F | 5’-AAGAGTTGTACCAGGGTGATGCCTT-3’ | Glycolysis |
|  | G6PD-R | 5’-GGCTCACTCTGCTTTCGGATGTCGT-3’ |  |
| *Hk1* | HK1-F | 5’-TCACCACTAGCGACGTAGCCGCCAT-3’ | Glycolysis |
|  | HK1-R | 5’-AGAACCGTCTACGCCAACTGTGGT-3’ |  |
| *Hk2* | HK2-F | 5’-GATGAGACCCTTCTGGAGATTTCTA-3’ | Glycolysis |
|  | HK2-R | 5’-CCATTGTCTGTCACCCTTACTCGGA-3’ |  |
| *Pfkfb3* | PFKFB3-F | 5’-TGTGAAGCAGTACAGCTCTTACAAC-3’ | Glycolysis |
|  | PFKFB3-R | 5’-GGTCGTCACAGACCGACTCGATGA-3’ |  |
| *Gapdh* | GAPDH-F | 5’-ATGACATCAAGAAGGTGGTGAAG -3’ | Glycolysis |
|  | GAPDH-R | 5’-TCCTTGGAGGCCATGTAGG-3’ |  |
| *Ldha* | LDHA-F | 5’-ACATTGTCAAGTACAGTCCACACTG-3’ | Glycolysis |
|  | LDHA-R | 5’-CACAGGCACACTGGAGTCGCCATGT-3’ |  |
| *Ldhb* | LDHB-F | 5’-TGGGAAAGTCTCTGGCTGATGAAC-3’ | Glycolysis |
|  | LDHB-R | 5’-GTTGACATTTCTCTGCACCAGGTTG-3’ |  |
| *Pdha1* | PDHE1α-F | 5’-TGTGACCTTCATCGGCTAGAA -3’ | TCA cycle |
|  | PDHE1α-R | 5’-TGATCCGCCTTTAGCTCCATC -3’ |  |
| *Cs* | CS-F | 5’-GGACAATTTTCCAACCAATCTGC-3’ | TCA cycle |
|  | CS-R | 5’-AGTCAATGGCTCCGATACTGC-3’ |  |
| *aKgd* | CS-F | 5’-GGACAATTTTCCAACCAATCTGC-3’ | TCA cycle |
|  | CS-R | 5’-AGTCAATGGCTCCGATACTGC-3’ |  |
| *Idh2* | IDH2-F | 5’-GCGCCACTATGCTGAGAAGAG -3’ | TCA cycle |
|  | IDH2-R | 5’-GGTCTGGTCACGGTTTGGAA -3’ |  |
| *Fh* | FH-F | 5’-GAATGGCAAGCCAAAATTCCTT-3’ | TCA cycle |
|  | FH-R | 5’-TCTTACGGTCTGAGCACCATAA -3’ |  |
| *Slc1a5* | SLC1a5-F | 5’-GCTGCTCATTCGTTTCTTCAACT-3’ | Glutaminolysis |
|  | SLC1a5-R | 5’-TGAAGAGGAAGTAGATGAGAGGCAG-3’ |  |
| *Glud1* | GLUD1-F | 5’-TACACGGAGGTTCACTATGGAGCT-3’ | Glutaminolysis |
|  | GLUD1-R | 5’-ATGTAAGAAGCCTCATTGATGAAGT-3’ |  |
| *Gls* | GLS -F | 5’-CACTCAAATCTACAGGATTGCGAAC-3’ | Glutaminolysis |
|  | GLS -R | 5’-CTTTCATATAACTCATCGATGTGTG-3’ |  |
| *Acly* | ACL-F | 5’-AGTTCTACGTGTGCATCTATGCT-3’ | Lipid synthesis |
|  | ACL-R | 5’-TCAGGTGCATGGACCAACAGGTGT-3’ |  |
| *Hmgcr* | HMGCR-F | 5’-GCCAGCTGGAGATCATGTGCTGCTT-3’ | Lipid synthesis |
|  | HMGCR-R | 5’-AGCTATCCAGCGACTATGAGCGTGA-3’ |  |
| *Fasn* | FASN-F | 5’-GGAGGTGGTGATAGCGGTAT-3’ | Lipid synthesis |
|  | FASN-R | 5’-TGGGTAATCCATAGAGCCCAG-3’ |  |
| *Dgat1* | DGAT1-F | 5’-TCCGTCCAGGGTGGTAGT-3’ | Lipid synthesis |
|  | DGAT1-R | 5’-GTGCCATCGTCTGCAAGATTC-3’ |  |
| *Elovl6* | ELOV6-F | 5’-GAAAAGCAGTTCAACGAGAACG-3’ | Lipid synthesis |
|  | ELOV6-R | 5’-AGATGCCGACCACCAAAGATA-3’ |  |
| *Mpc1* | MPC1-F | 5’-ATTGCTGCTATCAATGACATGAAGA-3’ | Mitochondrial pyruvate carrier |
|  | MPC1-R | 5’-AGCTACTTCGTTTGTTACATGGCAT-3’ |  |
| *Mpc2* | MPC2-F | 5’-ACTCATGGATAAAGTGGAGTTGTT-3’ | Mitochondrial pyruvate carrier |
|  | MPC2-R | 5’-CACAGTGGACTGAGCTGTGCTGAG-3’ |  |
| *Cpt1a* | CPT1a-F | 5’-AGATCAATCGGACCCTAGACACCAC-3’ | Fatty acid oxidation |
|  | CPT1a-R | 5’-AGAAGACCTTGACCATAGCCATCCA-3’ |  |
| *Cpt2* | CPT2-F | 5’-CAGACAGTGGCTACCTATGAATCCT-3’ | Fatty acid oxidation |
|  | CPT2-R | 5’-TGGTCAGCTGGCCATGGTATTTGGA-3’ |  |
| *Mcad* | Mcad-F | 5’-GAACACTTACTATGCCTCGATTGCA-3’ | Fatty acid oxidation |
|  | Mcad-R | 5’-AGCTATGATCAGCCTCTGAATTTGT-3’ |  |
| *Hadha* | Hadha-F | 5’-TCTGGGCCAACGACCAAATC-3’ | Fatty acid oxidation |
|  | Hadha-R | 5’-GGCTTIGATGAGATAAGGACAGC-3’ |  |
| *PPARa* | PPARa-F | 5’-AGAGCCCCATCTGTCCTCTC-3’ | Fatty acid oxidation |
|  | PPARa-R | 5’-ACTGGTAGTCTGCAAAACCAA-3’ |  |
| *PGC1a* | PGC1a-F | 5’-AGCCGTGACCACTGACAACGAG-3’ | Fatty acid oxidation |
|  | PGC1a-R | 5’-TGGTCAGCTGGCCATGGTATTTGGA-3’ |  |
| *PGC1b* | PGC1b-F | 5’-GGCAGGTTCAACCCCGA-3’ | Fatty acid oxidation |
|  | PGC1b-R | 5’-CTIGCTAACATCACAGAGGATATCTTG-3’ |  |
| *c-Myc* | c-Myc-F | 5’-AGACATGGTGAACCAGAGCTTCAT-3’ | Fatty acid oxidation |
|  | c-Myc-R | 5’-AGGCCAGCTTCTCCGAGACCAGCT-3’ |  |
| *Eif3s5* | EIF35S-F | 5′-CTGAGGATGTGCTGTCTGGGAA-3’ | Internal control |
|  | EIF35S-R | 5′-CCTTTGCCTCCACTTCGGTC-3′ |  |

*Glut1*, glucose transporter 1; *Glut4*, glucose transporter 1; *G6PD*, glucose-6-phosphate dehydrogenase; *HK1*, hexokinase 1; *HK2*, hexokinase 2; *PFKFB3*, 6-phosphofructo-2-kinase/fructose-2,6-biphosphatase; *Gapdh*, glyceraldehyde 3-phosphate dehydrogenase; *Ldha*, lactate dehydrogenase A; *Ldhb*, lactate dehydrogenase B; *Pdha1*, pyruvate dehydrogenase E1α; *Cs*, citrate synthase; *aKgd*, α-ketoglutarate dehydrogenase; *Idh2*, isocitrate dehydrogenase 2; *Fh*, fumarate hydratase; *Slc1a5*, solute carrier family 1 (neutral amino acid transporter) member 5; *Glud1*, glutamate dehydrogenase 1; *Gls*, glutaminase; *Acly*, ATP citrate lysae; *Hmgcr*, 3-hydroxy-3-methylglutaryl-CoA reductase; *Fasn*, fatty acid synthase; *Dgat1*, diacylglycerol O-acyltransferase 1; *Elovl6*, elongation of very long chain fatty acids protein 6; *Mpc1*, mitochondrial pyruvate carrier 1; *Mpc2*, mitochondrial pyruvate carrier 2; *Cpt1a*, carnitine palmitoyltransferase 1A; *Cpt2*, carnitine palmitoyltransferase 2; *Mcad*, **Medium-chain specific acyl-CoA dehydrogenase, mitochondrial;** *Hadha*, trifunctional protein alpha subunit; *PPARa*, peroxisome proliferator activated receptor alpha; *PGC1a*, PPARG coactivator 1 alpha; *PGC1b*, PPARG coactivator 1 beta; *Eif3s5* (Eukaryotic translation initiation factor 3, subunit 5) was used as an internal normalization control for real-time PCR.
